# Utilisation of Plant Food Waste

**DOI:** 10.3390/foods6060045

**Published:** 2017-06-14

**Authors:** Costas E. Stathopoulos

**Affiliations:** School of Science Engineering and Technology, Abertay University, Bell Street, Dundee DD1 1HG, UK; c.stathopoulos@abertay.ac.uk; Tel.: +44-1382-308481

This Special Issue “Utilisation of Plant Food Waste” of *Foods*, dedicated to Plant Food Waste Utilisation, showcases the attempts in labs across the world to try and reduce levels of global waste by recovering and re-using bioactive compounds. This approach towards a circular economy would not only reduce waste from the plant food industry but also potentially create and develop additional income streams for primary and secondary producers.

Work by a multinational team, comprising researchers from Algeria, Belgium and Italy, managed to produce and characterise thermostable and pH-stable polygalacturonase, using tomato pomace as a substrate. The production of this valuable biocatalyst was achieved by using an Aurobasidium pullanans strain isolated from Saharan soil in Algeria [1].

An Australia–UK collaboration has provided up-to-date information on using green aqueous extraction, combined with pre-treatment by UV-C irradiation, to recover phenolic compounds from lemon pomace. The recovered bioactives were also evaluated for their antioxidant capacity, compared with extracts obtained without pre-treatment. There are strong indications that, at an appropriate level, UV-C treatment at quite high doses can lead to increased yield of phenolic compounds [2].

Finally, in a thorough review, a team from Greece, Korea, Belgium and China have explored the potential of using plant food residues as a source of nutraceuticals and functional foods [3]. A number of bioactives are assessed, including pigments and antioxidants. The review explores current fruit and vegetable processing practices for recovery of high value products. A number of crops are examined in depth as potential sources of bioactive compounds, including all the main fruit crops in the world. This very well referenced review also includes an evaluation of the bioavailability of the recovered bioactive compounds.

We hope that readers will find this issue interesting, and that through similar issues at regular intervals this very important field of research will be further explored, conferring both environmental and financial benefits.
